# Circumcision and continuous antibiotic prophylaxis in preventing recurrent febrile urinary tract infection in boys with high-grade vesicoureteral reflux

**DOI:** 10.1016/j.jped.2026.101609

**Published:** 2026-09-21

**Authors:** Xiaoyu Tang, Liwen Sun, Xiangxiang Chen, Hui Wang, Jinxia Wang, Yan Chen, Yulin Kang, Yue Zhang, Yiqing Lyu, Fang Chen, Lijun Zhou, Yichen Huang

**Affiliations:** a Department of Urology, Shanghai Children's Hospital, School of Medicine, Shanghai Jiao Tong University, Shanghai, China; b Department of Nephrology and Rheumatology, Shanghai Children's Hospital, School of Medicine, Shanghai Jiao Tong University, Shanghai, China; c Clinical Research Unit, Shanghai Children's Hospital, School of Medicine, Shanghai Jiao Tong University, Shanghai, China; d School of Life Sciences and Biotechnology, Shanghai Jiao Tong University, Shanghai, China

**Keywords:** Vesicoureteral reflux, Children, Febrile urinary tract infection, Circumcision, Continuous antibiotic prophylaxis

## Abstract

**Objective:**

To evaluate the effectiveness of circumcision and continuous antibiotic prophylaxis (CAP) in preventing febrile urinary tract infection (fUTI) in boys with high-grade primary vesicoureteral reflux (VUR).

**Methods:**

This retrospective cohort included 114 boys younger than 5 years diagnosed with grades III–V primary VUR between June 2019 and June 2022. Patients were managed with observation (n = 14), CAP alone (n = 23), circumcision alone (n = 38), and combined CAP and circumcision (n = 39). The follow-up duration was 2 years. The incidence of fUTIs in each group was recorded, and associations between conservative management strategies and fUTI recurrence were analyzed.

**Results:**

15 (13.2%) children experienced recurrent fUTIs. Recurrence rates were 42.9% in the observation group, 30.4% in the CAP group, 5.3% in the circumcision group, and 0% in the CAP+ circumcision group. Circumcision-containing groups (circumcision alone and CAP + circumcision) had significantly lower recurrence rates than non-circumcision groups (observation and CAP alone) (p < 0.05). Multivariable Firth-penalized logistic regression confirmed circumcision as an independent protective factor against fUTI recurrence (aOR = 0.09, 95% CI: 0.01–0.98, p = 0.042), while CAP alone showed no significant protective effect (aOR = 0.45, 95% CI: 0.06–3.21, p = 0.421).

**Conclusion:**

Circumcision significantly reduced fUTI recurrence in boys with grade III–V primary VUR and may represent an important component of conservative management in this high-risk population, whereas the added value of CAP remains uncertain.

## Introduction

Primary vesicoureteral reflux (VUR) is a significant risk factor for febrile urinary tract infections (fUTI) in children [1,2]. Recurrent fUTIs are closely associated with progressive renal scarring, which is a crucial factor in developing end-stage renal disease (ESRD) [3]. Accordingly, prevention of fUTI recurrence remains a central therapeutic objective in the management of pediatric VUR [4].

Because VUR frequently resolves spontaneously, particularly in younger children, conservative management is a preferred approach. Common non-surgical strategies include observation, continuous antibiotic prophylaxis (CAP), and circumcision in boys [5]. CAP is the most widely recommended treatment [6]; however, concerns have been raised regarding its inconsistent efficacy in preventing recurrent UTIs and the potential risks of antimicrobial resistance, adverse effects, and long-term adherence.

Circumcision has been shown to reduce the incidence of UTIs in newborn boys [7] and to lower recurrence rates in children with other urological conditions, such as obstructive hydronephrosis and posterior urethral valves. Despite this evidence, circumcision remains an optional intervention in most clinical guidelines for VUR management [5,6,8]. Previous studies have reported a substantial reduction in UTI recurrence following circumcision compared with antibiotic prophylaxis alone [9]. Limited data also suggest that circumcision combined with CAP may further decrease infection risk, with lower urine culture positivity rates reported in grade I–III VUR [10].

However, clinical studies on the combined use of circumcision and CAP in preventing fUTI recurrence in high-grade VUR are limited. Therefore, this study retrospectively analyzed clinical data from boys younger than 5 years with high-grade III–V primary VUR treated at the institution between June 2019 and 2022 to evaluate the effectiveness of CAP, circumcision, and their combination in preventing recurrent fUTIs in this high-risk population.

## Methods

The retrospective cohort study included 114 boys younger than five years diagnosed with high-grade (III–V) primary VUR who received conservative management at the institution between June 2019 and June 2022. The study was approved by the Ethics Committee (approval number: 2018R022-E04), and all guardians provided informed consent.

### Study population

Patients were enrolled if they underwent voiding cystourethrography following either a first febrile urinary tract infection (fUTI) with abnormal urinary ultrasound findings or two fUTI episodes with normal ultrasound results, and were diagnosed with grade III–V VUR [11]. Febrile UTI was defined as a temperature ≥ 38 °C with a positive urine stick for nitrite and/or leukocyte esterase, in a urine sample collected by clean catch. Microbiological confirmation required a positive urine culture indicating the presence of at least one uropathogenic bacterium at a concentration ≥ 100,000 colony-forming units (CFU)/mL [12].

Exclusion criteria included: (1) secondary VUR (e.g., posterior urethral valve or neurogenic bladder); (2) associated urological conditions, including ureteropelvic junction obstruction, vesicoureteral junction stenosis, ectopic ureteral insertion, megaureter, renal hypoplasia, abnormal renal parenchymal echogenicity, duplicated collecting systems, horseshoe kidney, or other congenital malformations; (3) chromosomal abnormalities or other structural anomalies; and (4) incomplete follow-up or missing clinical data.

### Treatment strategies and follow-up

Baseline demographic and clinical variables, including age, blood pressure, and imaging findings at diagnosis, were collected. Treatment decisions were made by the patients’ guardians after comprehensive counseling regarding the potential benefits and risks of each conservative management option.

The patients were categorized into four groups: observation, continuous antibiotic prophylaxis (CAP) alone, circumcision alone, or combined CAP and circumcision treatment. Circumcision was performed after the diagnosis of VUR was confirmed and the urinary tract infection had been controlled. CAP was defined as continuous prophylactic antibiotic use for more than six months. CAP treatment consisted of oral nitrofurantoin, trimethoprim–sulfamethoxazole, or cephalosporins administered at one-third of the therapeutic dose once daily at bedtime. Antibiotic rotation every 1–3 months was guided by the nephrologist to minimize antimicrobial resistance, or prompted by minor adverse effects (e.g., gastrointestinal discomfort) and the results of urine cultures in cases of breakthrough fUTIs. During the regular outpatient or online consultations, CAP adherence was assessed through parental self-reporting. All patients completed follow-up every 6 months for a total of 2 years through outpatient clinic visits or online consultations to document the incidence of recurrent fUTI.

### Statistical analysis

Data distribution was assessed for normality. Normally distributed continuous variables were compared using the *t*-test, while non-normally distributed variables were analyzed using the Mann–Whitney *U* test. The Chi-square test or Fisher’s exact test was used for categorical variables. When overall group differences were significant, pairwise comparisons were performed with Bonferroni correction. Firth's penalized likelihood logistic regression was conducted to evaluate factors associated with fUTI recurrence. All statistical analyses were performed using IBM SPSS Statistics version 27.0, with a p value < 0.05 considered statistically significant.

## Results

### Baseline characteristics

A total of 114 boys were included in the analysis. The mean age at enrollment was 28.02 ± 17.67 months, with a mean height of 86.26 ± 17.25 cm and a mean weight of 13.16 ± 4.56 kg. Among these children, 31 (27.2%), 54 (47.4%), and 29 (25.4%) had grades III, IV, and V VUR, respectively. Unilateral VUR was present in 52 children (45.6%), whereas 62 (54.4%) had bilateral VUR.

Patients were allocated to four management groups: observation (n = 14), continuous antibiotic prophylaxis (CAP) alone (n = 23), circumcision alone (n = 38), and combined CAP+ circumcision (n = 39). Baseline demographic and clinical characteristics are summarized in Tables 1 and 2. Children who underwent circumcision received the procedure within 2.24±1.92 weeks after the initial diagnosis.

**Table 1:** Baseline of 114 children with vesicoureteral reflux.

	Observation	CAP	Circumcision	CAP + Circumcision	*P*-value
Total Number	14	23	38	39	
Age (months)	20.86 ± 15.36	32.04 ± 19.56	28.47 ± 17.63	27.79 ± 17.40	0.325
Height (cm)	78.71 ± 13.94	89.43 ± 17.62	86.58 ± 18.23	86.79 ± 17.20	0.327
Weight (kg)	11.44 ± 4.68	14.23 ± 4.97	12.91 ± 4.63	13.40 ± 4.21	0.328

CAP, continuous antibiotic prophylaxis.

**Table 2:** Vesicoureteral reflux (VUR) grades and laterality distribution in 114 boys with VUR.

Group	VUR Grade			VUR Laterality	
	III	IV	V	Unilateral	Bilateral
Observation	8	5	1	8	6
CAP	12	10	1	11	12
Circumcision	7	18	13	21	17
CAP + Circumcision	4	21	14	12	27
Total	31(27.2%)	54(47.4%)	29(25.4%)	52(45.6%)	62(54.4%)

Bilateral vesicoureteral reflux according to the side with the highest grade.

### Characteristics of recurrent fUTI cases

Recurrent febrile urinary tract infections occurred in 15 patients (13.2%) during follow-up. The mean age at recurrence was 12.80 ± 14.19 months. Among these patients, 9 (60.0%) had unilateral VUR and 6 (40.0%) had bilateral VUR. According to VUR grade, recurrent fUTIs were observed in 6 patients (40.0%) with grade III VUR, 7 (46.7%) with grade IV VUR, and 2 (13.3%) with grade V VUR.

### fUTI recurrence by treatment group

The recurrence rates of febrile urinary tract infection (fUTI) differed across the four treatment groups. Recurrent fUTIs were observed in 6 of 14 patients (42.9%) in the observation group, 7 of 23 (30.4%) in the CAP group, 2 of 38 (5.3%) in the circumcision group, and none of the 39 patients (0%) in the CAP + circumcision group. Overall differences in fUTI recurrence rates among the four groups were statistically significant (p < 0.001; Table 3).

**Table 3:** Treatment groups and febrile urinary tract infection (fUTI) recurrence in 114 male children with vesicoureteral reflux.

Group	Total,n	fUTI Recurrence, n(%)	No fUTI Recurrence, n(%)	*P*-value
Observation	14 ª	6(42.9%)	8(57.1%)	
CAP	23 **ª**	7(30.4%)	16(69.6%)	
Circumcision	38 **^b^**	2(5.3%)	36(94.7%)	<0.001
CAP + Circumcision	39 **^b^**	0(0.0%)	39(100.0%)	
Total, n(%)	114	15(13.2%)	99(86.8%)	

fUTI, febrile urinary tract infection; The letters “a” and “b” denote groups compared using Bonferroni pairwise comparisons. Groups with different letters show statistically significant differences.

Pairwise comparisons demonstrated significantly lower recurrence rates in the circumcision and CAP plus circumcision groups compared with the observation and CAP groups (Table 4). Among children who underwent circumcision, only two fUTI recurrences were observed, whereas no recurrences occurred in those receiving combined CAP and circumcision.

**Table 4:** Multivariable analysis of factors associated with recurrent fUTI using Firth's Penalized Logistic Regression.

Variables	Reference	Adjusted OR	95% CI	*P*-value
**Age (per month increase)**		0.58	0.21–1.52	0.271
**VUR Grade**	Grade III			
Grade IV		2.15	0.42–11.02	0.358
Grade V		1.82	0.23–14.36	0.569
**Laterality**	Unilateral			
Bilateral		2.96	0.45–19.58	0.259
**Treatment Group**	Observation			
CAP		0.45	0.06–3.21	0.421
Circumcision		0.09	0.01–0.98	0.042
CAP+Circumcision		0.02	0.00–1.86	0.089
**Subgroup analysis**	Circumcision alone			
CAP+Circumcision vs. Circumcision		0.68	0.05–9.25	0.462

Multivariable Firth-penalized logistic regression, adjusting for age, VUR grade, and laterality, showed that circumcision alone was independently associated with reduced fUTI recurrence (aOR = 0.09, 95% CI: 0.01–0.98, p = 0.042). CAP alone showed no significant protective effect (aOR = 0.45, 95% CI: 0.06–3.21, p = 0.421). Age, VUR grade, and laterality were not independent predictors (all p > 0.05). Compared with circumcision alone, the addition of CAP did not confer significant incremental benefit (aOR = 0.68, 95% CI: 0.05–9.25, p = 0.462).

## Discussion

Primary vesicoureteral reflux (VUR) affects approximately 1% (0.4–1.8%) of children and is frequently identified presenting with urinary tract infections (UTIs), particularly those with febrile episodes early in life [13,14]. Recurrent febrile UTIs (fUTIs) are strongly associated with renal scarring, which is a known risk factor for end-stage renal disease (ESRD) in children [15]. Consequently, reducing fUTI recurrence remains a central goal in the conservative management of VUR in young children.

Continuous antibiotic prophylaxis (CAP) has historically been the preferred conservative treatment for VUR, aiming to reduce fUTI recurrence through low-dose oral antibiotics. The RIVUR trial demonstrated an approximately 50% reduction in recurrent febrile or symptomatic UTIs (f/sUTI) among children receiving CAP compared with placebo [16]. However, the generalizability of these findings to boys remains uncertain, as only a small proportion of participants in RIVUR were male. In contrast, a meta-analysis by Williams et al. found no significant reduction in recurrent UTIs with long-term CAP compared with observation alone [17]. The reason for these discrepancies may partly lie in differences between the sexes. A large European multicenter study focusing on grade III–V VUR reported that CAP was effective in girls with high-grade reflux, whereas only a weak association was observed in boys, limited to those with grade IV VUR [18]. Consistent with these observations, the present study demonstrated no significant difference in fUTI recurrence between boys managed with CAP alone and those managed with observation, suggesting that the protective effect of CAP in boys with VUR may be limited.

Beyond concerns regarding efficacy, prolonged antibiotic exposure in early childhood has been associated with adverse outcomes, including increased risks of inflammatory bowel disease, autoimmune disorders, and metabolic alterations [[19], [20], [21]]. These concerns have prompted increasing interest in more selective, individualized prophylactic strategies. Recent work has explored risk-based approaches to CAP, including predictive modeling and machine learning to identify children most likely to benefit from antibiotic prophylaxis [2,22].

Circumcision represents another conservative option in the management of VUR in boys and has been associated with a reduced risk of UTIs in clinical trials [5,6,8]. Urinary tract infections in boys predominantly originate from the periurethral flora. Circumcision modifies this microenvironment, thereby reducing bacterial burden near the urethral meatus [23]. Neonatal circumcision has been associated with a substantial reduction in UTI risk, and similar protective effects have been reported in boys with congenital urological abnormalities, including hydronephrosis and posterior urethral valves [[24], [25], [26]]. In children with VUR, circumcision has been associated with lower rates of breakthrough UTIs, and prior studies have suggested additional benefit when combined with CAP, particularly in grade I-III reflux [9,10].

However, data addressing the combined effect of circumcision and CAP in boys with high-grade VUR remain limited. In the present study, circumcision was independently associated with a markedly reduced risk of recurrent fUTIs. Notably, recurrence rates were low among circumcised boys, and no recurrent fUTIs were observed in those receiving combined CAP and circumcision in the present study. These findings suggest that circumcision may be an important component of conservative management in boys with high-grade VUR. Although CAP didn’t add value in circumcised boys, its combination with circumcision had the lowest observed recurrence rate(0%). These results support an individualized approach to conservative therapy.

The lower enrollment of boys in large trials such as RIVUR may also reflect regional differences in circumcision practices, particularly in North America. Higher circumcision rates may reduce the incidence of symptomatic UTIs in boys, potentially leading to underdiagnosis of asymptomatic VUR. This observation provides indirect evidence that circumcision confers protection against UTIs in boys with VUR.

It should be emphasized that the present study assessed short-term infectious outcomes and didn’t evaluate the long-term natural history of VUR. Conservative management represents an important bridging strategy in VUR. Circumcision combined with CAP may serve as an important strategy during early childhood, aiming to prevent urinary tract infections and acute renal injury. For non-resolving cases beyond this period, particularly those approaching school age, the treatment strategy should be reassessed, with individualized decisions guided by risk factors such as bilateral disease, renal scarring, and bladder-bowel dysfunction to determine whether surgical intervention (e.g., ureteral reimplantation) is warranted.

Notably, even when VUR is effectively controlled, some patients remain at risk for long-term adverse outcomes. A recent transitional outcomes study on VUR patients who underwent surgical treatment highlighted that bilateral disease, bladder-bowel dysfunction, renal scarring, and older age at surgery (≥ 7 years) were associated with persistent risks of febrile UTI, lower urinary tract dysfunction, and pregnancy-related complications, even after VUR resolution [27]. This reveals that VUR management should extend beyond reducing fUTI recurrence to include individualized long-term risk stratification.

Several limitations of this study should be acknowledged. Its retrospective design, relatively small sample size, and two-year follow-up period may limit the generalizability of the findings. The wide age range of participants and the absence of standardized CAP regimens, including variation in antibiotic choice and rotation schedules, may have introduced additional heterogeneity. In addition, adherence to CAP was evaluated based on parental self-report rather than objective measures, such as pill counts, which may have introduced recall bias. Furthermore, long-term outcomes such as renal scar progression, lower urinary tract dysfunction, antimicrobial resistance, natural history of VUR and VUR resolution rates were not assessed. Prospective, multicenter studies with longer follow-up are needed to further clarify the role of circumcision, alone or in combination with CAP, in the conservative management of high-grade VUR in boys.

## Conclusion

In summary, this study demonstrates that circumcision is associated with a substantially reduced risk of recurrent febrile urinary tract infections in boys with primary high-grade vesicoureteral reflux. The combination of circumcision and continuous antibiotic prophylaxis was associated with the lowest recurrence rates during follow-up. The additional benefit of routine CAP in circumcised boys remains unproven. These findings support a more individualized approach to conservative management in this high-risk population.

## Funding

This work was supported by Shanghai Municipal Commission of Health and Family Planning (Grant No. 201840136) and 10.13039/501100004921Shanghai Jiao Tong University (Grant No. TMSK-2020-104).

## Authors’ contributions

Xiaoyu Tang, Liwen Sun, Lijun Zhou, and Yichen Huang contributed to the conception and design of the study, performed the statistical analyses, prepared the figures, and drafted and revised the manuscript. Xiangxiang Chen, Hui Wang, Jinxia Wang, Yan Chen, Yulin Kang, Yue Zhang, and Yiqing Lyu were responsible for data collection and manuscript revision. Xiaoyu Tang, Liwen Sun, Xiangxiang Chen, Hui Wang, Jinxia Wang, Yan Chen, Yulin Kang, Yue Zhang, Yiqing Lyu, Lijun Zhou, Yichen Huang, and Fang Chen contributed to patient follow-up. Lijun Zhou, Yichen Huang, and Fang Chen critically reviewed and edited the manuscript. All authors reviewed and approved the final version of the manuscript.

## Conflicts of interest

The authors declare no conflicts of interest.

## Data Availability

The data that support the findings of this study are available from the corresponding author upon reasonable request.
